# Riding toward inclusion: the journey of adapted cycling

**DOI:** 10.3389/fspor.2026.1770473

**Published:** 2026-04-15

**Authors:** Stephanie Sorraghan, Sally Logan, Valerie Watchorn, Dion Williams, Danielle Hitch, Joanne M. Watson, Kathryn Aedy, Therese Dogra, Pearse Fay, Matthew Haanappel, Aarti Shukla, Glen Lebeau, James Little, John McKenna, Kerry Townsend, Kate L. M. Anderson

**Affiliations:** 1 Occupational Therapy, School of Health and Social Development, Deakin University, Geelong, VIC, Australia; 2 School of Computing Technologies, STEM College, RMIT University, Melbourne, VIC, Australia; 3 Occupational Therapy, Allied Health, Western Health, St Albans, VIC, Australia; 4 Institute for Health Transformation, School of Health and Social Development, Deakin University, Geelong, VIC, Australia; 5 My Active Life, LEAG, Freedom Solutions Australia, Melbourne, VIC, Australia

**Keywords:** adapted cycles, assistive technology, disability, inclusive research, inclusive sport and recreation

## Abstract

**Introduction:**

This study examined how adapted cycles, as a form of assistive technology, support participation in sports and recreational activities for people with disability. Although physical activity is widely recognised for its benefits to physical health, psychological wellbeing, and social connectedness, people with disability continue to face significant barriers to meaningful inclusion in sport and recreation. Adapted cycles offer a means of addressing some of these barriers by enabling individuals to engage in cycling in ways that are responsive to their functional abilities and support needs.

**Methods:**

Using a qualitative research approach, this study drew on in-depth interviews with six participants, including children and adults, who had experience using adapted cycles across a range of contexts. This diversity allowed for a nuanced exploration of individual experiences. Data were analysed thematically to identify patterns related to engagement, perceived benefits, and factors influencing successful use.

**Results:**

Three key themes were identified: (1) navigating barriers and building pathways; (2) riding into wellbeing and inclusion; and (3) expanding horizons. Participants consistently reported improvements in physical health, including increased strength, endurance, and overall fitness. Many also described increased independence, noting that adapted cycles enabled them to access community spaces and engage in activities with reduced reliance on others. Importantly, these functional gains were valued for the ways in which they supported social inclusion. Participants described increased confidence and self efficacy, as well as a stronger sense of belonging when cycling with family members, friends, or within community groups.

**Discussion:**

Adapted cycles were perceived as facilitating social interaction, increasing visibility in public spaces, and challenging assumptions about disability and capability. The study also identified several contextual factors that shaped inclusive outcomes. These included access to customised and well-maintained equipment, quality assessment and selection services, accessible environments, and strong social support from peers, families, and organisations. Persistent barriers such as inaccessible transport and environments were noted to constrain inclusion opportunities. Overall, the findings contribute to broader discussions on inclusive recreation and assistive technology by demonstrating that the value of adapted cycling lies not in participation itself, but in its capacity to foster meaningful social inclusion and enhance wellbeing.

## 1 Introduction

Cycling is one of the most popular forms of physical recreation in Australia (1). Engagement in cultural life, including sports and leisure, is a fundamental human right protected by the United Nations Convention on the Rights of Persons with Disabilities (2). Despite this, a recent international synthesis (3) reported that people living with disability are between 16%–62% less likely to meet physical activity guidelines than people without disability. While these data primarily come from high-income countries in the Northern Hemisphere, the finding is consistent with the lower rates of participation in sport and physical leisure by people with disability reported elsewhere, including Chile (4) and our own context of Australia (5). Factors contributing to lower participation rates include limited access to suitable and desirable sports activities and equipment for people with disability (6, 7).

Addressing this gap, adapted cycles (as defined in ISO 9999:2022) encompass a wide range of assistive technologies designed to meet the needs of cyclists with diverse mobility and balance challenges (8). For instance, foot-propelled tricycles with extra-wide bases support cyclists who experience balance difficulties, recumbent trikes offer back and neck support for cyclists with reduced core strength, while tandem cycles enable a support person to cycle alongside someone with vision impairment or co-ordination difficulties. Additional adaptations like foot straps, trunk supports, headrests, seat belts, and electric motors also allow people to cycle with greater ease and independence (9). Diverse examples and images of these adaptations can be seen in Australia's National Equipment Database (10). In this paper, we use *adapted cycles* to refer to cycles that are purpose-built or modified to enable riding for people who cannot use a standard bicycle safely or comfortably (9).

A recent scoping review by Mosser, Norcliffe and Kruse (11) found that adapted cycling can yield a range of positive health impacts, including improvements in cardiovascular fitness, muscle strength, and overall physical wellbeing. Several studies have highlighted physical benefits for specific groups of cyclists. For instance, children with cerebral palsy have demonstrated improvements in gross motor skills and postural ability (12–14). A broader systematic review of literature relating to children with disability and cycling by Thevarajah et al. (15) also concluded adapted cycling can positively affect body structures and function. However, the authors noted that additional high-quality studies are needed to strengthen confidence in these findings.

Adapted cycling may also enhance activity performance, independence, and social participation for people with disability. Fletcher et al. (14) found that adapted cycling enabled children with disability to ride independently with their peers and family while multiple case studies have illustrated how adapted cycling can expand a person's social networks (13, 14, 16, 17) and enable participation in sport and adventure activities (18). Despite these findings, limited attention has been given to *how* adapted cycling impacts cyclists’ identity or quality of life, or which factors influence these broader outcomes.

Research into the experiences of people who use adapted cycles and their supporters highlight key factors that influence outcomes gained. Ensuring that the chosen cycle meets, and continues to meet, the individual user's needs is critical, with past research indicating that bespoke fitting services reportedly reduced fear of riding (17), mitigated pain or injuries from riding (18), and increased riding function (7, 18, 19). Comprehensive assessment, accompanied by training for users and their supporters, is also highly valued (14, 17). Consequently, while cycles themselves represent valuable ‘hard technology’, supporting ‘wrap-around’ services (20), also referred to as soft technology (21), are equally vital to achieving effective outcomes.

Environmental factors also influence both the practicality and enjoyment of cycling. Known facilitators in the physical environment include access to an appropriate adapted cycle and opportunities to ride within a safe, stimulating and accessible setting (13, 22, 23). Social factors, including adequate time to practise (13) and a supportive social environment (17), further enhance the cycling experience. Together, the interacting effects of personal, activity, environmental, and technology factors reflect previously established models of assistive technology selection and use [e.g., the Human Activity Assistive Technology (HAAT) model (24)] and recreational participation more broadly (25) demonstrating that holistic approaches to selection and outcome evaluation in adapted cycling are needed.

Adapted cycling sits at the intersection of disability sport/recreation, assistive technology, and disability studies: fields that offer unique and sometimes competing views on participation, inclusion, and wellbeing. While prior work has described both barriers and benefits to adapted cycling, there is ongoing debate about what ‘inclusion’ means in this context, or how broader factors shape what is possible. There is also increasing recognition that inclusive recreation research is strengthened when lived expertise shapes the design and interpretive frame. Contributing to this conversation, our framing question for this research was: ‘How do people with disability and their supporters experience life with an adapted bike?’

## 2 Methodology

### 2.1 Study context

This study formed part of a broader project investigating the My Active Life program (Freedom Solutions Australia). Guided by people with lived experience of disability, My Active Life aimed to increase sport and recreation participation across Australia through a range of community-based initiatives. This included the national Freedom Wheels program that co-designs adapted cycles for children and adults with disability (26).

In Australia, responsibility for inclusive recreation is distributed across multiple sectors. As a sport, cycling is governed through the Federal Australian Sports Commission (Sport Australia) and through the peak body AusCycling. Adapted cycling aligns with both Australia's Inclusive Sport Framework (27) and AusCycling's remit for para-cycling and participation pathways (28). Access to adapted cycling as a disability support is also supported by Australia's Disability Strategy 2021–2031 (29), but implementation and resourcing varies by jurisdiction and policy climate. Australia's National Disability Insurance Scheme (NDIS) also provides individualised funding for supports to take part in social and recreation activities (30). Nonetheless, anecdotal evidence suggests that service delivery often relies on hybrid models combining formal supports with philanthropy and volunteer labour (e.g., skilled volunteers engineering and fitting the cycles as part of community assistive-technology organisations). This complexity is commensurate with global patterns in assistive technology service provision that demand a cross-sectoral approach (31).

This study comprised an exploratory qualitative investigation of experiences of adapted cycling for people who had used the Freedom Wheels adapted cycling service. The study received ethical approval from Deakin University Human Research Ethics Committee (Project ID: HEAG-H 188 2021).

### 2.2 Study design and methodological orientation

The research followed an inclusive research paradigm (32) collectively led by academics, industry advisors and a Living Experience Advisory Group (LEAG). LEAG members were people with disability with an interest in inclusive recreation. They did not have clinical or research experience, but contributed experience as carers, disability service providers, or professional advocates. The research team was intentionally multidisciplinary (comprising occupational therapy, exercise science/public health, and critical disability studies) and several researchers also had lived experiences of disability and/or informal care. We explicitly acknowledge that our LEAG and research team could not represent all perspectives across disability, geography, cultural background, and cycling contexts. Accordingly, we engaged in ongoing reflexive practice – regularly asking ‘who is not at the table?’ and using inclusive research guidelines to inform protocol decisions and interpretation (33, 34).

The project was designed within an interpretivist, constructivist paradigm, recognising that participants’ accounts and researcher interpretations are shaped by social context and embodied experience. Our approach acknowledges the complex interactions between material phenomena (e.g., pain, strength, morphology) and social and environmental constructs (e.g., access, stigma, body image) (35). The project was sensitised by a rights-based lens and theoretical frameworks from our respective disciplines, including the Principles of Universal Design (36), the Model of Human Occupation (37), the HAAT model of assistive technology (24), Martin Ginis and colleagues’ socioecological model of physical activity participation (3), and the International Classification of Functioning, Disability and Health (ICF) (38).

LEAG members were consulted at every study stage, contributing to project design (e.g., informing inclusive and accessible recruitment approaches and methods of data collection), undertaking collaborative data analysis, and co-authoring project outputs. LEAG members were paid for all work plus any travel expenses. The GRIPP2-LF reporting checklist (39) supported transparent reporting all patient and public involvement (Supplementary Appendix A). Reflections on, and lessons learned, from this inclusive research partnership are also addressed in Anderson et al. (40).

### 2.3 Recruitment

Participants were recruited via the partner organisation Freedom Solutions Australia. Recruitment information was also disseminated via the research team's professional networks. Participants were required to: 1) have engaged with the Freedom Wheels service between May 2021 and March 2022, 2) be able to participate in English, 3) have lived experience of disability, and 4) be involved in sport and recreation activities. Participants who did not meet these criteria were excluded.

Our recruitment approach aimed for breadth of experience rather than statistical representation. We did not implement selective or stratified sampling; however, recruitment materials and data collection approaches were designed to encourage diversity across age, ability, and cycling experience. A custom recruitment form provided study information in multiple formats (plain written language, EasyRead and video) and offered accessible consent options, including supported decision-making for children or people with significant cognitive disability and their guardians (41). The recruitment form also gathered information about participants’ access needs and interview preferences.

### 2.4 Data collection

To maximise accessibility and respect participant preference, we adopted a bricolage approach to data collection (42) by offering the following options for participation: a) synchronous spoken interviews (in-person/online/phone); b) email responses; and c) arts-based expression (photographs or drawings) that participants could submit and discuss (43). This approach, guided by our LEAG, honoured diversity in communication preferences, access needs, and logistical constraints. Five participants engaged through spoken interviews and one provided an email response; no participants submitted photos or drawings.

Interviews were conducted online via Zoom by qualitative researchers DW and KA, who are both disability inclusion specialists, and student researcher SS. Each semi-structured interview addressed 15 questions across three general topics: 1) their bike and how they received it; 2) bike use and satisfaction; and 3) experiences in sports and recreation since receiving the bike. LEAG members advised on enhancing question accessibility and re-centring interviews around the guiding question: ‘What’s life like with a bike?’. The full interview schedule is provided in Supplementary Appendix B.

Interviews ranged from 47 to 86 min. All were audio recorded and transcribed verbatim. To affirm ongoing consent, participants were invited to review their transcript and no amendments or withdrawals were requested. Interviewers recorded reflexive field notes documenting their perceptions and contextual observations (44), for discussion during team-based analysis.

### 2.5 Participants

Six participants took part; three children and three adults. Participants resided in three Australian states (New South Wales, Western Australia, Victoria), mostly in metropolitan areas (*n* = 5). Most identified as female (*n* = 4). Due to young age or communication disability, five participants were assisted by a supporter: where supporters provided input, they were prompted to reflect on the cyclist’s experiences rather than their own.

### 2.6 Data analysis

Reflexive thematic analysis (RTA) (33) was selected to support a rich, interpretive analysis of meanings around adapted cycling, aligning with our constructivist orientation. Analysis followed Braun and Clarke's recursive phases (33, 45), beginning with the team undergoing a process of familiarisation with the data. Primary coders DW, KA, VW, and SS read the transcripts and written materials, reviewed field notes, and recorded memos capturing assumptions, uncertainties, and initial interpretative hunches. Data were then coded line-by-line in NVivo, capturing both explicit descriptive content (what participants said, e.g., ‘Family recreational activities’), and interpretative elements (e.g., ’Sense of belonging and normalcy’). DW and SS then grouped codes into candidate themes.

Early themes, illustrated with aggregated vignettes and selected quotes, were reviewed and refined by the wider team including the LEAG. Differences in interpretation were used to clarify meaning and theme boundaries, and to consider missing perspectives. Themes and subthemes were then named and iteratively refined through team discussion and written draft reviews. LEAG members helped to contextualise findings and queried interpretations that did not resonate with their own observations. For example, while preparing for a joint conference presentation, LEAG members helped to recentre several themes on the transformative role of adapted cycling across participants’ lives. A sample of the relationship between raw data, codes, and themes is illustrated in Table 1.

**Table 1:** Sample of theme generation

Overarching theme	Subtheme	Code	Quote
Riding into Wellbeing and Inclusion	Feeling Proud, Confident, and Happy	Adapted bike provides sense of freedom through independence	‘It's just nice to be out, and people see me and it's just, it's nice to be on the bike and no one, you're on your own, and, years ago I had a horse, and it was like that independence, you know, go off and ride your horse, and no one can tell me anything, 'This is the same' (Cyclist Five)
		Sense of belonging and normalcy	‘I think it helps reinforce the fact that they [Cyclist Two] can do what everybody else does.’ (Supporter Two)
		Adapted bike can facilitate independent participation in daily family activities	‘We take it to [shops], and I also ride it there.’ (Cyclist Three)
		Opportunity for family recreational activities	‘Where we went [on holidays], they had bike paths which were massive, and we rode every day. My daughter loves it.’ (Cyclist Five)

Our coding approach was primarily inductive. Throughout, we reflected on sensitising discipline theories to critique and sharpen our interpretations without forcing data into a pre-set framework. However, these theories are not strongly foregrounded in the analysis or discussion, as participants’ accounts emphasised a dynamic, journeyed experience of adapted cycling that was poorly reflected in existing static models of participation.

The study is reported in line with RTA reporting guidelines (46). Quality strategies reflect our interpretivist epistemology: quality was supported through continuous reflexive practice (33), interpretive accountability to lived expertise through LEAG involvement (47), and transparency in analytic and processes claims (46).

## 3 Findings

Analysis of the data generated three themes: (1) navigating barriers and building pathways; (2) riding into wellbeing and inclusion; and (3) expanding horizons.

### 3.1 Navigating barriers and building pathways

This theme described participants’ motivations toward adapted cycling and key factors shaping their engagement. It comprised four subthemes: ‘personal capabilities and motivations’; ‘assistive technology as an enabler’; ‘the physical environment’; and ‘the support environment’.

#### 3.1.1 Personal capabilities and motivations

Participants described both motivations and challenges that shaped the journey into adapted cycling. Initial uncertainty, anxiety, and concerns about safety were common. One child described drawing on personal courage to overcome their fear of cycling, noting that riding now makes them feel ‘brave like a lion.’ (Cyclist Three). Their advice to other children was ‘don't be scared’. Cyclists were attuned to their own developing capabilities and valued being heard, as seen in Cyclist Five's recount of her assessment:

‘When I went there, there was three [cycles] I tried. And I was right, [the] tricycle was the best for me, because of getting on. They were able to put special toe things on that hold up [the pedal], because for me, as soon as you get off, the foot things fall down, and I can't lift them up! Such a simple little ‘nothing’ thing. But my husband’s like, ‘why do you need that?’ I'm like, ‘because I can't do it!’…’.

Participants also emphasised personal agency in their adapted cycling journey. Supporter Four described waiting to see if Cyclist Four chose her wheelchair or bike before an outing, ‘…so we know, okay, she just wants a break today, or she actually wants to take a bike.’

#### 3.1.2 Assistive technology as an enabler

Adapted cycle features were central to enabling participation and required careful tailoring to cyclist's physical, cognitive, emotional, and sensory capabilities. Adjustability was highly valued for extending usability over time. Supporter One described the cycle as ‘… really built for life in terms of the measurements and the ability to adjust settings,’ while Supporter Two noted: ‘… if he does continue to improve his strength, it is easy enough to swap the pedals over.’

Personalised and functional accessories enhanced participation, safety and enjoyment. Cyclist Five noted noting how, with added postural support and seating stability: ‘My balance…I would sit on the bike and I feel safe. Whereas if I'm standing, I fall over. So, on the bike I'm safer than I am standing.’ Push bars enabled steering assistance, as Supporter Two noted: ‘… I can ride behind him or get out behind him and push him you know.’ Independence could also be achieved through the addition of outrigger training wheels that were ‘really wide and they’re fixed so there's no give.’ (Supporter Three).

Person-centred features enabled access to previously inaccessible terrains, such as kerbs sand, and grass. Supporter Four described travelling where ‘… there's going to be a bit of rough terrain, taking her bike means it can go up kerbs, it can go onto sand, it can go onto grass, when her wheelchair can't.’ However, the larger size and weight of an adapted cycle could also present barriers to transportation and independent use, with Supporter One noting that ‘it's usually me who's left to try and lift this bike’, and Supporter Three reflecting that wide wheelbases made footpaths ‘a bit of a disaster.’ Similarly, Supporter Four added:

‘It is heavy, it’d be better if it was lighter because it is quite difficult to get into the back of a car… not everybody has a ute [utility vehicle] or a four-wheel-drive. They don’t have that room, so they might not have the option to just chuck the bikes in the car and take them to the park.’

Gear use and electric motors were perceived as valuable for navigating hills. Supporter Two found that ‘[Cyclist Two] is still using mainly the lower gears when he's going downhill… so we’ve still got a lot of strength to build up.’ Cyclists Five's access to an electric motor was described as essential: ‘because I've got the electric, we're in a hilly area, I can go out the front of my house and go down the hill and come back and go up the hill into the driveway.’

Finally, accessorising the cycle added personal salience: common accessories included baskets for toys or drinks, or bells for fun and practicality. Aesthetic design also enabled the expression of individuality and was often a conversation starter. As Supporter Four observed:

‘When it comes to the disability field, you kind of get stuck, ‘this is what's going to suit your disability, and this is what's going to help you’, but you have no say in what it looks like…sometimes they just want the pretty pink bike.’

#### 3.1.3 The physical environment

Physical environments shaped cycle use and influenced the functionality of cycle features. For example, narrow and often obstructed footpaths forced Cyclist Three to ride on the road. These potential barriers were better accounted for in the case of Cyclist One: ‘…they [staff] asked her where her preference was to ride the bike, which would determine what kind of wheels they put on the bike.’ Transport infrastructure was also critical, particularly for families transporting cycles:

‘… so we got a van. A wheelchair van with a lift, and the bike fits nicely on the wheelchair lift, and nicely in the back of the van. That would be a negative with the bike if you didn't have that sort of system, because …, you can't put it on a bike carrier or any of those sort of things ..’ (Supporter Two).

The design of local environments influenced choices of riding location, perceived safety and independence. Unsafe cycling tracks and traffic hazards prompted some families to seek alternatively spaces that originally planned:

‘We tried originally her going around the main track. That was too difficult to try …[and] meant that she could easily swerve into traffic… Or she could go into a normal, you know, regular cyclist…, so we then went to the kid's bike track area and that's where she uses it.’

Environmental accessibility extended beyond cycling specific facilities to the broader infrastructure of sport and recreational spaces – as Supporter Four highlighted: ‘… is there a ramp, is there steps, is there a toilet available?’ Other aspects of infrastructure, such as the availability of adult sized change tables, were necessary for ‘privacy [and] dignity’ (Supporter Four). However, in some cases adapted cycles helped participants overcome general environmental barriers to recreation:

‘It's not just the activity itself [people] are having trouble with, it's the actual access to the activity…We've got a park that's literally two streets away from our house. But there's no footpath leading to the park, and the park is completely covered in sand. Whenever we walk past, the kids are like, ‘can we go to the park?’ And I'm like, well, there's no footpath to the park. But the other day we were actually able to go to the park because we took the bike.’ (Supporter Four).

#### 3.1.4 The support environment

Supporters played a central role in enabling people with disability to participate in adapted cycling. In particular, families were seen to play active roles in building cyclist confidence, teaching riding skills, supervising safe riding, and managing equipment logistics. As Cyclist Five explained: ‘I had no confidence and even now, it took me a little bit to go out, every day I want to go out, but I wouldn't go unless my husband was here.’ Nonetheless, this role could be challenging for cyclists and families, making professional support essential:

‘… They have got to be prepared to, and have the capacity to lift this heavy bike, and many are not keen to do that. …In an ideal world if they could send somebody out to help train the person on the bike that would be brilliant, because otherwise it's left to the family to do that, or to source somebody who can do that.’ (Supporter One).

Participants reported that the process of acquiring an adapted cycle involved assessment, trial, training, applying for funding, and review and that staff expertise, inclusive attitudes, inter-service collaboration, timely delivery, and follow-up appointments were integral to a positive experience. As Supporter Six stated, ‘it was good to trial a couple of different bikes to see what suited Cyclist Six the best’. Supporters praised organisational staff for their knowledge, transparency, and responsiveness during the assessment process. For instance, Supporter Three described the staff as ‘…clearly very knowledgeable - they put her on a couple of different bikes. Just from watching a walk up the stairs they can pretty well tell you know what she was going to need’. Similarly, staff expertise was noted to positively influence the selection of cycle features during the assessment process, ensuring these were tailored to participants’ preferences and abilities. As Supporter Four described, ‘They had to adjust some of the settings, they had to make the seat lower, bring it forward a bit. Just so that it fits her properly when her legs are going around in the circle’. Participants commended the way that staff privileged lived-experience input during these decision-making processes: ‘Yeah they were absolutely fabulous, both in terms of showing [Cyclist One] a variety of options and empowering her to make the decision.’ (Supporter One).

Participants noted that assessments and trials were typically conducted in purposefully designed environments and that colourful murals and obstacle courses created perceptions of inclusion and fun.:

‘… they open these doors and you’re like in this magical painted.. All the walls have got these sort of cartoon murals on them and then, the floor is customised design… And it was just an incredibly inclusive and positive and fun experience. And it sort of instantly gave her a sense of confidence on the bike.’ (Supporter One)

Obstacle courses used during trials were also noted to build participants’ skill and confidence:

‘… it's like it's a zigzag track that goes around all the bikes and he rode for 5–10 min and didn't run into any of the bikes, which I found amazing, that was good. And then what, when you got your bike, well they did they did the same thing didn’t they?’ (Supporter Two).

However, a need for ongoing support and education beyond the trial period was also evident:

‘She [trial staff member] pressed some button and it braked it, and I don’t know what that is, I’ve tried! So, every time we come out, still, we park it facing up the hill, because if I get off it, and I can’t put it in brake and we’re on a slide, it's gone-ski, gone… Would’ve just given me confidence, definitely, if they came [to provide on-road training]. But no, I was so lucky to have it, so I wasn’t complaining.’ (Cyclist Five)

### 3.2 Riding into wellbeing and inclusion

Reflecting outcomes gained from adapted cycling, this theme emerged from three subthemes: ‘physical health benefits’; ‘feeling proud, confident, and happy’; and ‘enhanced social opportunities’.

#### 3.2.1 Physical health benefits

Participants consistently reported that using an adapted cycle provided valuable physical exercise and contributed to improved health. Cyclist Five reflected on personal progress noting that: ‘We're trying to build my strength’ and ‘my fitness is much better.’ Similarly, Supporter Three commented that: ‘… [Cyclist Three has] picked up strength in her legs really quickly to the point where she doesn't really need us pushing very much. Often, it's more holding her back, so she doesn't take off down a hill’. Similar benefits were also noted for Cyclist Two:

‘… If he continues to increase… his strength, the school has a bike club. And they ride once a week, and they go out through the bush. And we were thinking next year, he will definitely be strong enough to go ride with those kids.’ (Supporter Two).

#### 3.2.2 Feeling proud, confident, and happy

Beyond physical benefits, participants and supporters described how cycling fostered a sense of achievement, boosted confidence, and created feelings of enjoyment.

Findings highlighted that the ability and freedom to ride independently was a valued outcome of using adapted cycles, for example, giving Cyclist One ‘.. an ability to literally move on her own’. Adapted cycles also supported independence in family responsibilities, such as enabling Cyclist Four ‘to go pick up her sister from school’ or enabling Cyclist Three ride to the shops. Beyond daily tasks, participants celebrated the opportunities generated for enjoyable recreation. Cyclist Two enjoyed the simple pleasure of cycling close to home: ‘on the road, on *our* road’, while the adapted cycle had enriched family holidays for Cyclist Five and her daughter: ‘they had bike paths which were massive, and we rode every day. My daughter loves it.’

Reflecting that being able to cycle is an expected societal norm, supporters commented that the adapted cycle ‘has given [Cyclist Six] the confidence to ride a bike, something that he didn’t think would ever happen.’ and ‘I think it helps reinforce the fact that they [Cyclist Two] can do what everybody else does.’ Cyclist Three echoed this:

‘It's just nice to be out, and people see me and it's just, it's nice to be on the bike and no one, you're on your own, and, years ago I had a horse, and it was like that independence, you know, go off and ride your horse, and no one can tell me anything, this is the same’.

#### 3.2.3 Enhanced social opportunities

A prominent theme was enhanced social inclusion with family, friends, and communities: For Supporter One, cycling ‘[is] always combined with being with family, with being outdoors and fresh air… with accessing the community.’ Supporter Four similarly observed that cycling had ‘also helped with that the inclusivity as well, being part of the family and not being excluded because of her disability. She's able to ride a bike like the rest of her sisters now, which is fantastic.’ Likewise, Cyclist Two enjoyed riding alongside family members who were active in sports, recalling that: ‘…everyone was riding when I was doing the bike riding.’

Adapted cycling also strengthened peer inclusion. Supporter Three described the shift in participation and social engagement:

‘Every day after school there's a group of her friends who go and play at the park across the road and they bring their scooters and bikes and she will generally just stand with the adults, because she can't she hasn't been able to ride a bike, she's too scared to ride on a scooter and not stable enough anyways, so she's kind of excluded sort of pretty badly from her peer group because she just can't keep up with them, whereas now she actually can.’

The adapted cycle was also reported to create opportunities for community engagement and inclusion. Cyclist Three's experience highlighted the social benefits of moving independently in public:

‘[in the pram] she reaches up and pulls the cover down and she's not really, kind of actually engaging with anyone in the community. Whereas when she's on her bike people say hi to her and people say ‘oh it's such a great bike’ and she's getting a lot more interaction from just the general public.’ (Supporter Three)

Similarly, Supporter Six and Cyclist Five shared experiences of community curiosity and social interaction: ‘he gets people saying, ‘cool bike’…’ (Supporter Six); ‘and when I've been out, I put learner plates on it. People say, ‘oh, I want to have a go, can you take my kids?’ I'm like ‘hell no, I'm enjoying myself thanks!’ You know, I love it!’ (Cyclist Five).

### 3.3 Expanding horizons

The personal transformations people experienced in their health, wellbeing and social opportunities, and the resultant impacts on their self- and social- perception, ultimately expanded their horizons for inclusion. This final theme addresses how adapted cycles created a gateway to inclusion’ for participants and unpacks the factors that shaped this outcome.

#### 3.3.1 Gateway to inclusion

Adapted cycling was perceived as a gateway to broader inclusion, providing participants with the confidence and capability to engage in additional sports, recreational pursuits, and community activities. For many, increased confidence from cycling acted as a catalyst for trying new experiences. Since receiving their adapted cycle, participants reported pursuing a variety of activities, including horse riding (Cyclist One), competitive cycling and bush cycling (Cyclist Two), and gymnastics (Cyclist Three). Cyclist Five highlighted how the confidence gained from cycling had expanded their personal engagement in fitness more broadly: ‘Oh! My confidence! I now go and do personal training twice a week.’ For individuals with acquired impairments, adapted cycling also rekindled connections to previous recreational interests. Cyclist Five described how renewed engagement in cycling inspired the consideration of new challenges: ‘I'm swimming, and straight away when I got the bike I started to wonder if I could do a triathlon again, you know, I can't run but maybe someone can help me.’

#### 3.3.2 Factors impacting inclusion

Supporters described their responsibilities as parents or guardians. For example, often needing to ‘advocate why [Cyclist Six] should be included’ in sport and recreational activities (Supporter Six). Beyond advocacy, inclusion was enhanced when others, such as peers, demonstrated supportive attitudes and knowledge of disability, fostering patience, encouragement, and a sense of belonging. Conversely, differences in capability from peers could limit opportunities for participation, and sometimes necessitated intervention from supporters:

‘Her friends without disability tend to exclude her when it comes to sport, physical recreation, or even bowling… because she can’t really keep up with them… It becomes an activity that's done more within an environment with the family rather than in a general social context.’ (Supporter One).

The attitudes and skills of coaches and organisational staff were noted as key enablers to inclusion. Coaches with education or personal experience of disability were perceived as more supportive, whereas those lacking understanding could inadvertently contribute to perceptions of exclusion. Supporter Two described this challenge: ‘The coach doesn't understand how to deal with a child [with disability] … they get excluded because they can't include them because they don't know what to do to include.’ To address this issue, participants emphasised the need for greater formal education to equip coaches with strategies for inclusion. As stated by Supporter Two ‘We don't make any effort in sport… to really educate people’.

Importantly, participants described inclusion in sport and recreation as more than just participation; it also encompassed feelings of normalcy, belonging, and meaningful connection. Cyclist Five reflected on inclusion as feeling ‘normal’ and like you are a ‘part of something,’ while Supporter Six emphasised skill development and contribution: ‘…to fully engage in sport and recreation in a meaningful way, be competitive, build skills, and become ‘an asset to the team, community, or sport.’’

## 4 Discussion

While the benefits of adapted cycling for physical health have been long documented (11–14, 16, 17, 48), until now few studies had addressed broader implications for family wellbeing or social inclusion. This study explored the lived experiences of people with disability using adapted cycles, with findings highlighting their profoundly positive impact and revealing both anticipated outcomes and novel insights. Benefits included enhanced functional mobility, confidence, and community participation. At the same time, several factors, such as the large weight and size of adapted cycles, limited transport options, and inaccessible environments, were found to restrict or hinder people's cycling experiences and the outcomes they could achieve. These insights extend what is known about adapted cycling and contribute to broader discussions on inclusive recreational opportunities.

To consider these findings, the discussion below is organised around two themes: (1) physical, social and technological influences; and (2) enhancing personal wellbeing and social inclusion.

### 4.1 Physical, social, and technological influences

Findings from the current study indicate that physical, social, and technological aspects of the environment affect the functionality of assistive technology and strongly shape outcomes related to adapted cycle use. Consistent with this finding, the ICF (38) conceptualises disability as an outcome of a complex and unique interaction between an individual's health condition, personal factors, and environmental influences. Recognising that environments shape both opportunity and empowerment provides an essential foundation for the themes that follow, which consider the influence of physical, social, and technological contexts and the deeper importance of genuine inclusion.

#### 4.1.1 Physical environment

This study reinforces and extends upon previous research showing that a person's experience of adapted cycling is greatly influenced by natural and built environments, particularly in terms of perceived safety and cycling quality (22, 49, 50). Consistent with previous research, participants highlighted the importance of well-designed infrastructure, noting that traffic volume, inaccessible paths and road surface quality significantly influenced their cycling experience. However, this study also revealed new insights, such as the restrictive effect of ground surfaces like grass and sand in recreational areas and the benefits of wider cycling paths that better accommodate adapted cycles.

Environmental barriers in the natural and built environments continue to limit the participation of people with disability in cycling and outdoor recreational activity more broadly (51, 52). It is therefore essential that these barriers are recognised and addressed by those involved in planning, policy, and design of recreational environments. Universal design offers a practical framework for mitigating these barriers (51, 53). By enhancing accessibility and usability for all people, universal design encourages designers to go beyond minimum standards and legislation, creating buildings and facilities that are flexible and accommodating of human diversity, including differences in ability, age, size, language, gender, and cultural background (36, 54, 55).

In their scoping review on adapted outdoor physical activities, Derakhshan et al. (51) utilise the Principles of Universal Design (36) to conceptualise how the usability and accessibility of natural and built environments can be enhanced. For instance, by applying Principle 1 – Equitable Use, Principle 7 – Size and Space for Approach and Use, Principle 6 – Low Physical Effort, and Principle 4 – Perceptible Information (36), designers are encouraged to: design pathways that provide sufficient space for adapted cycles; select ground surfaces that support wheeled mobility; provide maps of accessible cycling tracks, accessible bathroom facilities, secure storage, and larger parking bays for adapted cycles.

#### 4.1.2 Social environment

Cycling can be intimidating when learning to ride for the first time, and reassurance and support may be needed to overcome this. Participants in this study reflected on their early apprehensions and emphasised the importance of support from family and other supporters as well as comprehensive, context-specific interventions that build confidence, particularly regarding road safety. Reflecting the needs of all cyclists, not just those with disability, our findings reinforce that training and practice plays a critical role in promoting cyclist safety and confidence (13, 22, 50, 56) and that this should occur in both controlled and uncontrolled environments and evolve as a cyclist's skills develop with time.

Peers and fellow cyclists also influence people's experiences of cycling as a recreational activity, with supportive and patient individuals enhancing engagement and enjoyment (51, 57, 58). Noting that segregated activities can limit social inclusion (59), participants in this study expressed a preference to participate in sport and recreation activities alongside people without disability. Free community-based cycling and integrated programs, designed for participants with and without disability, have been found to foster inclusion by promoting interaction and support and by offering shared experiences (57, 58). Feeling ‘normal’ and able to engage alongside others was central to participants’ sense of inclusion and motivation to continue. Where integrated opportunities are limited, mainstream programs must prioritise supportive attitudes and disability education among staff and participants to enhance inclusive experiences.

The social environment, including the attitudes and behaviours of coaches, instructors, and peers, strongly shapes experiences of inclusion. When reflecting on their experiences of participating in cycling and other recreational activities, participants in this study perceived coaches as both facilitatory and challenging depending on their knowledge, approach, and support. Those with disability-inclusion education were perceived as better able to foster inclusion, aligning with evidence that leaders who understand disability can model inclusive behaviour and provide appropriate support within the context of sport and recreation (57, 58, 60). Conversely, sport and recreation coaches lacking awareness may unintentionally contribute to exclusion. These findings emphasise the importance of increasing disability awareness and education for coaches and others involved in sport and recreation to promote inclusive participation.

#### 4.1.3 Adapted cycles as assistive technology

Assistive technology is widely recognised as a key enabler of inclusion for people with disability across sport and recreation, education, and the broader community (31, 61, 62). Limited access to adapted cycles continues to restrict participation for many people with disability (6, 7). Given the substantial physical, social and wellbeing benefits associated with adapted cycling, it is critical that funding providers and policy makers recognise and respond to the need for appropriate, fit-for-purpose assistive technology.

Another important barrier occurs when equipment is not well-matched to an individual's abilities, goals, or cycling environment. When an adapted cycle does not align with a person's strength, balance, cognitive needs, or activity needs it can create new demands, such as increased fatigue, difficulty manoeuvring, safety concerns or frustration. These mismatches can limit confidence, reduce enjoyment, and undermine the very outcomes the technology is intended to support. This finding aligns with earlier research showing that people may reduce their use of assistive technology or abandon it altogether when it introduces new challenges or fails to meet their personal or functional needs (31, 63).

To reduce abandonment and optimise participation, assistive technology must be selected and customised with close attention to a person's goals, intended use, and potential barriers (31, 64). However, equitable access remains a persistent issue. High costs, limited funding pathways, and low awareness of available options continue to constrain opportunities for people with disability to obtain the adapted cycles and technologies they require (51, 65). To address these barriers, policymakers and service providers should prioritise wrap-around support, such as training, maintenance, and information about assistive technology options, while also strengthening funding mechanisms that reduce cost burdens and expand equitable access. Noting Australia's fragmented policy and funding ecology around adapted cycling, integrated review and co-ordination of existing infrastructure are also likely required.

### 4.2 Enhancing personal wellbeing and social inclusion

Adapted cycling can catalyse meaningful personal and social change. At the personal level, participants in this study reported that adapted cycling enhanced their sense of agency, creating opportunities to make autonomous choices, build confidence, and improve overall wellbeing. At the social level, adapted cycling was perceived to strengthen social capital by facilitating connections and prompting participation in broader community activities and networks. Here, a critical finding emerged that whilst adapted cycling increased participation, more significantly, it created pathways to authentic social inclusion, characterised by belonging and mutual recognition within community contexts.

#### 4.2.1 Personal wellbeing and social connection

Whilst previous research has documented the physical health improvements associated with adapted cycling in people with disability (12–14, 16, 17, 48), this study illuminates psychological and emotional dimensions of adapted cycling participation. Participants reported enhanced personal wellbeing, alongside feelings of pride, confidence, and happiness. More broadly, these emotional and psychological gains reflect broader improvements in personal agency, defined as an individual's belief that they can act meaningfully upon their world and pursue their aspirations (66). This study suggests that adapted cycling serves as a vehicle for cultivating personal agency, fostering confidence, sense of accomplishment, and emotional wellbeing. Beyond known physical benefits, this study shows that adapted cycles can become vehicles for personal agency, accomplishment, and emotional wellbeing. Further research exploring the reciprocal relationship between agency development and sustained participation in sports and recreation, including adapted cycling, is warranted.

Previous researchers have described cycling as a positive social experience (14, 16), shared activity (22, 23, 50, 67), or social confidence booster (50, 68), but little attention has been given to *why* cycling is such a powerful social mediator. This study suggests that adapted cycling strengthens community connections by acting as a *social catalyst*. Participants reported that adapted cycles frequently served as conversation starters and that cycling facilitated connections with peers and community members that extended beyond the activity itself into broader community networks. Some cyclists also reported feeling empowered to pursue new recreational goals following their initial cycling triumphs. Broadly, these findings align with Labbé et al. (69) who identified social benefits of adapted recreational activities for people with disability, including opportunities to meet new people and socialise with friends and family. However, further research is needed to examine the types of and impacts of social networks created through adapted cycling – including those beyond the recreation sphere.

#### 4.2.2 Social inclusion

The ICF (38) explicates how a health condition and the experience of disability can impact a person's participation, and the right to participation in recreational, leisure and sport is identified in Article 30 of the United Nations Convention on the Rights of Persons with Disabilities (2). However, findings from this study emphasise that participation does not automatically equate to inclusion.

In disability literature, inclusion is broadly considered in two ways; a state of being, and something people can practice (70). Inclusion as a state of being refers to when people feel included within their chosen activities and are allowed into society in a way equal to those around them. Inclusion can also be practiced, meaning people are able to actively make choices and display actions that enable others, such as people with disability, to be included. Hocking (70) suggests that inclusion does not merely mean being present or participating within activities in society. Instead, action can be taken to enable people to feel included within activities, and without such action, people may not experience inclusion as a state of being.

Participants in this study discussed feeling included when they could experience feelings of belonging, being competitive, building connections, and increasing skills during cycling. This supports earlier findings by McConkey et al. (58), whereby inclusion was perceived as meaning ‘togetherness,’ and experienced when people with disability were equally involved in sporting activities, made friends, and were given assistance to successfully participate. This is further supported by the socioecological framing of Martin Ginis et al. (3) who argue that the subjective experience of participation cannot be understood separately from its composite aspects of autonomy, belongingness, challenge, engagement, mastery, and meaning.

Whilst an individual may be able to participate in sport and recreation, as reflected in the ICF (38), this may not lead to the outcomes valued and sought after by people with disability. The finding of inclusion and the meaning attached to it from the participants in this study potentially challenges the notion of participation in the ICF as simply ‘involvement in a life situation’ (38). Instead, further research is needed to critique international disability frameworks and question if these concepts should aspire to capture and demand that the deeper meaning and multifaceted concept of inclusion is a more representative expectation.

For the recreation sector, these findings point to the need for a deliberate shift from delivering *accessible* programs to cultivating *inclusive* experiences. This requires practitioners, organisations, and governing bodies to attend not only to physical access and participation metrics, but also the social and relational conditions that facilitate a sense of belonging, competence, and connection. At a practical level, this means embedding inclusion into program design and evaluation, investing in ongoing training (for cyclists and their supporters) that focuses on autonomy, and creating environments where people with disability can engage as athletes, peers, and community members rather than passive recipients of support. Without these shifts, opportunities for recreation may be present, but the promise of social inclusion will remain unrealised.

### 4.3 Limitations of the research

This study has several limitations that should be considered when interpreting findings. As the study was situated within an Australian context, findings may not be transferable to other regions or socio-political environments. Time constraints and recruitment delays resulted in a small sample size, and findings may not reflect experiences of a larger group of cyclists. In addition, five of the six participants attended with a supporter, and although supporters were asked to share participants’ perspectives rather than their own, some responses may nevertheless reflect supporters’ experiences rather than those of people with disability themselves. Participants also provided subjective accounts of past experiences, which may influence accuracy of recollections. Finally, collection of more detailed demographic data regarding participants’ experiences of disability, neurodivergence, and other aspects of identity may have strengthened interpretation of the findings. Despite these limitations, the study maintains methodological rigour and offers valuable insights into the adapted cycling experiences of people with disability.

## 5 Conclusion

Life with a bike proved to be a transformative experience for the cyclists and supporters who shared their stories. Our study finds that, whilst an individual may be able to ‘participate’ in sport and recreation as defined by the ICF (38), this may not lead to the outcomes valued and sought after by people with disability. Instead, participants framed true cycling inclusion as a complex and embodied experience of belonging, connection, and agency, with impacts extending well beyond the bike path.

Our study contributes to ongoing conversations about inclusive recreation by showing that adapted cycling is best understood as a dynamic, socio-technical and relational phenomenon (11, 13, 14, 16, 17, 23). Consistent with socioecological accounts of disability (38), physical activity participation (25), and assistive technology adoption (20, 21, 31) findings from this research suggest that cycling inclusion is not determined by individual capacity alone, but is instead shaped by interacting factors, including technology characteristics, wrap-around services, cycling environments, social supports, and policy. Echoing the HAAT model (24), our findings highlights that the ‘fit’ of an assistive technology item (such as an adapted cycle) extends beyond technical performance to include fit for context, comfort, identity, and aesthetic. For those providing adapted cycling services, these findings underscore the need for holistic tailoring of technology, technical, and relational expertise (23), and iterative long-term support. For the recreation sector, the findings indicate a need to move beyond a simple ‘access’ mindset toward intentional designs that enable agency, dignity, and social connection (56). Intersecting domains, including the built and natural environment, workforce capacity, and recreation policy and funding, will be essential considerations for this work. Finally, for researchers and evaluators, our findings signal the importance of outcome frameworks that capture psychosocial and identity-related impacts alongside functional outcomes, and that attend to participation as a lived, evolving process.

Ultimately, our findings show that adapted cycling has potential to advance wellbeing and social inclusion for many people with disability. Realising this potential will require coordinated, long-term investment in the supports and environments that make adapted cycling safe, accessible, and sustainable. Done well, this would support people with disability to cycle with confidence, and leverage possibilities far beyond the bike path.

## Data Availability

The datasets presented in this article are not readily available because Consent was not gained to share data collected in this study. Requests to access the datasets should be directed to valerie.watchorn@deakin.edu.au.
