# Supplementary material for: Riding toward inclusion: the journey of adapted cycling
Source: Front Sports Act Living. 2026 Apr 15;8:1770473. doi: 10.3389/fspor.2026.1770473 (PMC13125089; doi:10.3389/fspor.2026.1770473)
Supplement: Supplementary file 1 [file Table1.docx]

**Supplementary Appendix A – GRIPP2-LF Reporting Checklist (1)**

| **Section and Topic** | | **Item** | | **Reported on Page No.** |
| --- | --- | --- | --- | --- |
| *Section 1: Abstract of paper* | | | | |
| 1a: Aim | | Report the aim of the study | | Details are reported in section 1 and 2.1; elaborated in: https://freedomsolutions.org.au/wp-content/uploads/2025/08/DeakinUniversityResearchReportonMyActiveLifeProgram.pdf |
| 1b: Methods | | Describe the methods used by which patients and the public were involved | | Details are reported in section 2; elaborated in: Anderson, K., Watchorn., V., Williams, D., Logan, S., Watson, J., Hitch, D., Fay, P., & Aedy, K. (2023). Impact of and factors supporting collaboration and participatory design. In An investigation of inclusive sport and recreation in the My Active Life program. (pp. 51 – 60). Deakin University. https://freedomsolutions.org.au/wp-content/uploads/2025/08/DeakinUniversityResearchReportonMyActiveLifeProgram.pdf |
| 1c: Results | | Report the impacts and outcomes of PPI in the study | | Please see: Anderson, K., Watchorn., V., Williams, D., Logan, S., Watson, J., Hitch, D., Fay, P., & Aedy, K. (2023). Impact of and factors supporting collaboration and participatory design. In An investigation of inclusive sport and recreation in the My Active Life program. (pp. 51 – 60). Deakin University. https://freedomsolutions.org.au/wp-content/uploads/2025/08/DeakinUniversityResearchReportonMyActiveLifeProgram.pdf |
| 1d: Conclusions | | Summarise the main conclusions of the study | | Details are reported in section 5; elaborated in https://freedomsolutions.org.au/wp-content/uploads/2025/08/DeakinUniversityResearchReportonMyActiveLifeProgram.pdf |
| 1e: Keywords | | Include PPI, “patient and public involvement,” or alternative terms as keywords | | Keywords added to this publication. |
| *Section 2: Background to paper* | | | | |
| 2a: Definition | | Report the definition of PPI used in the study and how it links to comparable studies | | Note, we are using the term Inclusive Research in this paper in line with disability focused research. Details are reported in section 2.2; elaborated in Anderson, K., Watchorn., V., Williams, D., Logan, S., Watson, J., Hitch, D., Fay, P., & Aedy, K. (2023). Impact of and factors supporting collaboration and participatory design. In An investigation of inclusive sport and recreation in the My Active Life program. (pp. 51 – 60). Deakin University. https://freedomsolutions.org.au/wp-content/uploads/2025/08/DeakinUniversityResearchReportonMyActiveLifeProgram.pdf |
| 2b: Theoretical underpinnings | | Report the theoretical rationale and any theoretical influences relating to PPI in the study | | Details are reported in section 2; Anderson, K., Watchorn., V., Williams, D., Logan, S., Watson, J., Hitch, D., Fay, P., & Aedy, K. (2023). Impact of and factors supporting collaboration and participatory design. In An investigation of inclusive sport and recreation in the My Active Life program. (pp. 51 – 60). Deakin University. Elaborated in https://freedomsolutions.org.au/wp-content/uploads/2025/08/DeakinUniversityResearchReportonMyActiveLifeProgram.pdf |
| 2c: Concepts and theory development | | Report any conceptual models or influences used in the study | | Details are reported in section 2; elaborated in https://freedomsolutions.org.au/wp-content/uploads/2025/08/DeakinUniversityResearchReportonMyActiveLifeProgram.pdf |
| *Section 3: Aims of paper* | | | | |
| 3: Aim | | Report the aim of the study | | Details are reported in section 1; elaborated in https://freedomsolutions.org.au/wp-content/uploads/2025/08/DeakinUniversityResearchReportonMyActiveLifeProgram.pdf |
| *Section 4: Methods of paper* | | | | |
| 4a: Design | | Provide a clear description of methods by which patients and the public were involved | | Details are reported in section 2; elaborated inhttps://freedomsolutions.org.au/wp-content/uploads/2025/08/DeakinUniversityResearchReportonMyActiveLifeProgram.pdf AND https://blogs.deakin.edu.au/inclusivehealth/wp-content/uploads/sites/423/2024/02/Guidelines-for-Inclusive-Research-1.pdf |
| 4b: People involved | | Provide a description of patients, carers, and the public involved with the PPI activity in the study | | People with lived experience of disability, some of which also act as carers, also industry advisors were involved in this study. |
| 4c: Stages of involvement | | Report on how PPI is used at different stages of the study | | Details are reported in section 2; elaborated in https://freedomsolutions.org.au/wp-content/uploads/2025/08/DeakinUniversityResearchReportonMyActiveLifeProgram.pdf AND https://blogs.deakin.edu.au/inclusivehealth/wp-content/uploads/sites/423/2024/02/Guidelines-for-Inclusive-Research-1.pdf |
| 4d: Level or nature of involvement | | Report the level or nature of PPI used at various stages of the study | | PPI level and nature are reported in section 2. Additional detail can be found in https://freedomsolutions.org.au/wp-content/uploads/2025/08/DeakinUniversityResearchReportonMyActiveLifeProgram.pdf AND https://blogs.deakin.edu.au/inclusivehealth/wp-content/uploads/sites/423/2024/02/Guidelines-for-Inclusive-Research-1.pdf |
| *Section 5: Capture or measurement of PPI impact* | | | | |
| 5a: Qualitative evidence of impact | If applicable, report the methods used to qualitatively explore the impact of PPI in the study | | | Please see: Anderson, K., Watchorn., V., Williams, D., Logan, S., Watson, J., Hitch, D., Fay, P., & Aedy, K. (2023). Impact of and factors supporting collaboration and participatory design. In An investigation of inclusive sport and recreation in the My Active Life program. (pp. 51 – 60). Deakin University. https://freedomsolutions.org.au/wp-content/uploads/2025/08/DeakinUniversityResearchReportonMyActiveLifeProgram.pdf |
| 5b: Quantitative evidence of impact | If applicable, report the methods used to quantitatively measure or assess the impact of PPI | | | N/A |
| 5c: Robustness of measure | If applicable, report the rigour of the method used to capture or measure the impact of PPI | | | Please see: Anderson, K., Watchorn., V., Williams, D., Logan, S., Watson, J., Hitch, D., Fay, P., & Aedy, K. (2023). Impact of and factors supporting collaboration and participatory design. In An investigation of inclusive sport and recreation in the My Active Life program. (pp. 51 – 60). Deakin University. https://freedomsolutions.org.au/wp-content/uploads/2025/08/DeakinUniversityResearchReportonMyActiveLifeProgram.pdf |
| *Section 6: Economic assessment* | | | | |
| 6: Economic assessment | If applicable, report the method used for an economic assessment of PPI | | | N/A |
| *Section 7: Study results* | | | | |
| 7a: Outcomes of PPI | Report the results of PPI in the study, including both positive and negative outcomes | | | Please see: Anderson, K., Watchorn., V., Williams, D., Logan, S., Watson, J., Hitch, D., Fay, P., & Aedy, K. (2023). Impact of and factors supporting collaboration and participatory design. In An investigation of inclusive sport and recreation in the My Active Life program. (pp. 51 – 60). Deakin University. https://freedomsolutions.org.au/wp-content/uploads/2025/08/DeakinUniversityResearchReportonMyActiveLifeProgram.pdf |
| 7b: Impacts of PPI | Report the positive and negative impacts that PPI has had on the research, the individuals involved (including patients and researchers), and wider impacts | | | Please see: Anderson, K., Watchorn., V., Williams, D., Logan, S., Watson, J., Hitch, D., Fay, P., & Aedy, K. (2023). Impact of and factors supporting collaboration and participatory design. In An investigation of inclusive sport and recreation in the My Active Life program. (pp. 51 – 60). Deakin University. https://freedomsolutions.org.au/wp-content/uploads/2025/08/DeakinUniversityResearchReportonMyActiveLifeProgram.pdf |
| 7c: Context of PPI | Report the influence of any contextual factors that enabled or hindered the process or impact of PPI | | | Please see: Anderson, K., Watchorn., V., Williams, D., Logan, S., Watson, J., Hitch, D., Fay, P., & Aedy, K. (2023). Impact of and factors supporting collaboration and participatory design. In An investigation of inclusive sport and recreation in the My Active Life program. (pp. 51 – 60). Deakin University. https://freedomsolutions.org.au/wp-content/uploads/2025/08/DeakinUniversityResearchReportonMyActiveLifeProgram.pdf |
| 7d: Process of PPI | Report the influence of any process factors, that enabled or hindered the impact of PPI | | | Please see: Anderson, K., Watchorn., V., Williams, D., Logan, S., Watson, J., Hitch, D., Fay, P., & Aedy, K. (2023). Impact of and factors supporting collaboration and participatory design. In An investigation of inclusive sport and recreation in the My Active Life program. (pp. 51 – 60). Deakin University. https://freedomsolutions.org.au/wp-content/uploads/2025/08/DeakinUniversityResearchReportonMyActiveLifeProgram.pdf |
| 7ei: Theory development | Report any conceptual or theoretical development in PPI that have emerged | | | Please see: Anderson, K., Watchorn., V., Williams, D., Logan, S., Watson, J., Hitch, D., Fay, P., & Aedy, K. (2023). Impact of and factors supporting collaboration and participatory design. In An investigation of inclusive sport and recreation in the My Active Life program. (pp. 51 – 60). Deakin University. https://freedomsolutions.org.au/wp-content/uploads/2025/08/DeakinUniversityResearchReportonMyActiveLifeProgram.pdf |
| 7eii: Theory development | Report evaluation of theoretical models, if any | | | N/A |
| 7f: Measurement | If applicable, report all aspects of instrument development and testing (eg, validity, reliability, feasibility, acceptability, responsiveness, interpretability, appropriateness, precision) | | | Please refer to section 2.2 and 2.4. Also please see https: Anderson, K., Watchorn., V., Williams, D., Logan, S., Watson, J., Hitch, D., Fay, P., & Aedy, K. (2023). Impact of and factors supporting collaboration and participatory design. In An investigation of inclusive sport and recreation in the My Active Life program. (pp. 51 – 60). Deakin University. AND https://blogs.deakin.edu.au/inclusivehealth/wp-content/uploads/sites/423/2024/02/Guidelines-for-Inclusive-Research-1.pdf |
| 7g: Economic assessment | Report any information on the costs or benefit of PPI | | | Refer to section 2.2. |
| *Section 8: Discussion and conclusions* | | | | |
| 8a: Outcomes | | | Comment on how PPI influenced the study overall. Describe positive and negative effects | Please see: Anderson, K., Watchorn., V., Williams, D., Logan, S., Watson, J., Hitch, D., Fay, P., & Aedy, K. (2023). Impact of and factors supporting collaboration and participatory design. In An investigation of inclusive sport and recreation in the My Active Life program. (pp. 51 – 60). Deakin University. https://freedomsolutions.org.au/wp-content/uploads/2025/08/DeakinUniversityResearchReportonMyActiveLifeProgram.pdf |
| 8b: Impacts | | | Comment on the different impacts of PPI identified in this study and how they contribute to new knowledge | Please see: Anderson, K., Watchorn., V., Williams, D., Logan, S., Watson, J., Hitch, D., Fay, P., & Aedy, K. (2023). Impact of and factors supporting collaboration and participatory design. In An investigation of inclusive sport and recreation in the My Active Life program. (pp. 51 – 60). Deakin University. https://freedomsolutions.org.au/wp-content/uploads/2025/08/DeakinUniversityResearchReportonMyActiveLifeProgram.pdf |
| 8c: Definition | | | Comment on the definition of PPI used (reported in the Background section) and whether or not you would suggest any changes | N/A |
| 8d: Theoretical underpinnings | | | Comment on any way your study adds to the theoretical development of PPI | Please see: Anderson, K., Watchorn., V., Williams, D., Logan, S., Watson, J., Hitch, D., Fay, P., & Aedy, K. (2023). Impact of and factors supporting collaboration and participatory design. In An investigation of inclusive sport and recreation in the My Active Life program. (pp. 51 – 60). Deakin University. https://freedomsolutions.org.au/wp-content/uploads/2025/08/DeakinUniversityResearchReportonMyActiveLifeProgram.pdf |
| 8e: Context | | | Comment on how context factors influenced PPI in the study | Please see: Anderson, K., Watchorn., V., Williams, D., Logan, S., Watson, J., Hitch, D., Fay, P., & Aedy, K. (2023). Impact of and factors supporting collaboration and participatory design. In An investigation of inclusive sport and recreation in the My Active Life program. (pp. 51 – 60). Deakin University. Please see: https://freedomsolutions.org.au/wp-content/uploads/2025/08/DeakinUniversityResearchReportonMyActiveLifeProgram.pdf |
| 8f: Process | | | Comment on how process factors influenced PPI in the study | Please see: Anderson, K., Watchorn., V., Williams, D., Logan, S., Watson, J., Hitch, D., Fay, P., & Aedy, K. (2023). Impact of and factors supporting collaboration and participatory design. In An investigation of inclusive sport and recreation in the My Active Life program. (pp. 51 – 60). Deakin University. https://freedomsolutions.org.au/wp-content/uploads/2025/08/DeakinUniversityResearchReportonMyActiveLifeProgram.pdf |
| 8g: Measurement and capture of PPI impact | | | If applicable, comment on how well PPI impact was evaluated or measured in the study | Please see: Anderson, K., Watchorn., V., Williams, D., Logan, S., Watson, J., Hitch, D., Fay, P., & Aedy, K. (2023). Impact of and factors supporting collaboration and participatory design. In An investigation of inclusive sport and recreation in the My Active Life program. (pp. 51 – 60). Deakin University. https://freedomsolutions.org.au/wp-content/uploads/2025/08/DeakinUniversityResearchReportonMyActiveLifeProgram.pdf |
| 8h: Economic assessment | | | If applicable, discuss any aspects of the economic cost or benefit of PPI, particularly any suggestions for future economic modelling. | N/A |
| 8i: Reflections / critical perspective | | | Comment critically on the study, reflecting on the things that went well and those that did not, so that others can learn from this study | Please see: Anderson, K., Watchorn., V., Williams, D., Logan, S., Watson, J., Hitch, D., Fay, P., & Aedy, K. (2023). Impact of and factors supporting collaboration and participatory design. In An investigation of inclusive sport and recreation in the My Active Life program. (pp. 51 – 60). Deakin University. https://freedomsolutions.org.au/wp-content/uploads/2025/08/DeakinUniversityResearchReportonMyActiveLifeProgram.pdf |

**References**

1. Staniszewska S, Brett J, Simera I, Seers K, Mockford C, Goodlad S, et al. GRIPP2 Reporting checklists: tools to improve reporting of patient and public involvement in research. *Br Med J*. (2017) 358:j3453. doi: 10.1136/bmj.j3453
