# Supplementary material for: Riding toward inclusion: the journey of adapted cycling
Source: Front Sports Act Living. 2026 Apr 15;8:1770473. doi: 10.3389/fspor.2026.1770473 (PMC13125089; doi:10.3389/fspor.2026.1770473)
Supplement: Supplementary file 2 [file Datasheet1.docx]

**Supplementary Appendix B – Interview Schedule**

For this first section let's have a chat about your bike and how you got it:

- Tell me about your bike
- Why did you decide to get a bike?
- How did you find out about Freedom Wheels?
- Tell me about your experience with the assessment and trial process (Clinic)
  - *Probe for: Did you get what you needed/wanted from the assessment and trial?*
  - *What did you/did you not like about the assessment and trial?*
  - *Is there anything that could be improved about the assessment and trial?*
- Is there anything you would like to tell us about the Freedom Wheels service (for example, paperwork, getting funding, or having your bike delivered?)

For this section let’s talk about what it has been like since you got your bike?

- How are you using your bike?
  - *Where? How often? Who with?*
- How happy are you with your bike?
  - Why are / are you not happy with your bike?
  - Does your bike do what you want it to do?
  - Are there any features of your bike you wish could change?
- Do you think you will you keep using it?

For this part of the interview, let’s talk about your experiences in sports, recreation and leisure since you got your bike.

- What does sports, recreation and leisure look like for you, now you have your bike?
- What did sports, recreation and leisure look like before you got your bike?
- What does inclusion mean to you in relation to sport, recreation and leisure?
  - What sort of things help you feel included?
  - What sort of things make you feel excluded?
- What else has changed since you got your bike?

That is the end of the interview. Is there anything else you would like to add?
